# Supplementary material for: The influence of leprosy-related clinical and epidemiological variables in the occurrence and severity of COVID-19: A prospective real-world cohort study
Source: PLoS Negl Trop Dis. 2021 Jul 28;15(7):e0009635. doi: 10.1371/journal.pntd.0009635 (PMC8351963; doi:10.1371/journal.pntd.0009635)
Supplement: S1 STROBE Checklist — (DOC) [file pntd.0009635.s001.doc]

**The influence of leprosy-related clinical and epidemiological variables in the occurrence and severity of COVID-19: A prospective real-world cohort study.**

STROBE Statement—Checklist of items that should be included in reports of ***cohort studies***

|  | Item No | Recommendation | Corresponding text |
| --- | --- | --- | --- |
| **Title and abstract** | 1 | (*a*) Indicate the study’s design with a commonly used term in the title or the abstract | A prospective real-world cohort study. |
|  |  | (*b*) Provide in the abstract an informative and balanced summary of what was done and what was found | Abstract |
| Introduction | | |  |
| Background/rationale | 2 | Explain the scientific background and rationale for the investigation being reported | Because leprosy is a mycobacterial infection that is highly dependent on Th1/Th2 modulation, and leprosy prophylaxis involves BCG vaccination in some countries, leprosy-endemic regions are interesting settings for assessments of the effects of these variables on the occurrence of COVID-19 in leprosy patients. |
| Objectives | 3 | State specific objectives, including any prespecified hypotheses | The main objective of this study was to assess the influence of leprosy-related clinical and epidemiological variables as risk/protective factors for the occurrence and severity of COVID-19. |
| Methods | | |  |
| Study design | 4 | Present key elements of study design early in the paper | We performed a 14-month prospective real-world cohort study in which the main exposure was defined as 2 previous BCG vaccinations, and the main outcome was defined as COVID-19 detected by reverse transcription polymerase chain reaction (RT-PCR). |
| Setting | 5 | Describe the setting, locations, and relevant dates, including periods of recruitment, exposure, follow-up, and data collection | From March to May 2020, patients were enrolled consecutively at the Leprosy Outpatient Service of Brasília University Hospital, University of Brasília, Brazil. |
| Participants | 6 | (*a*) Give the eligibility criteria, and the sources and methods of selection of participants. Describe methods of follow-up | This site is responsible for supporting more than 60% of all leprosy patients in the region, which has a population of more than 3 million people, and for performing differential diagnoses in all cases referred from secondary and primary healthcare facilities. |
|  |  | (*b*) For matched studies, give matching criteria and number of exposed and unexposed | Not applicable |
| Variables | 7 | Clearly define all outcomes, exposures, predictors, potential confounders, and effect modifiers. Give diagnostic criteria, if applicable | We performed a 14-month prospective real-world cohort study in which, for better statistical follow-up, the main exposure was defined as 2 previous BCG vaccinations, and the main outcome was defined as COVID-19 detection by reverse transcription–polymerase chain reaction (RT-PCR). BCG status (0, 1 or 2 doses) and leprosy exposure status (active disease, household contact of a patient with leprosy or control) were defined as secondary exposures of interest. Time at risk was calculated from data enrolment to the end of follow-up, censoring or the onset of COVID-19. |
| Data sources/ measurement | 8* | For each variable of interest, give sources of data and details of methods of assessment (measurement). Describe comparability of assessment methods if there is more than one group | At the first interview, patients were invited to participate in the study, and clinical information was collected. Patients were evaluated monthly at face-to-face medical consultations, via assessments of electronic medical files or telephone to collect information related to the target outcomes, including COVID-19 occurrence and the presence of complications. |
| Bias | 9 | Describe any efforts to address potential sources of bias | For the main exposure, a hierarchical model in which the occurrence of COVID-19 was defined as the main outcome was constructed. |
| Study size | 10 | Explain how the study size was arrived at | We arbitrarily considered that 50% of patients with fewer than two BCG vaccinations would develop COVID-19 and that only 35% of patients with two vaccinations would develop symptomatic SARS-CoV-2 infection (1-alpha = 95; 1-beta = 80%; relative size cases/controls = 1). |
| Quantitative variables | 11 | Explain how quantitative variables were handled in the analyses. If applicable, describe which groupings were chosen and why | For the main exposure, secondary exposure and possible confounders, relative risks (RRs), hazard ratios (HRs), log-rank tests and survival evaluations by the Kaplan-Meier method were performed to detect any possible influence on the occurrence of COVID-19. |
| Statistical methods | 12 | (*a*) Describe all statistical methods, including those used to control for confounding | a hierarchical model in which the occurrence of COVID-19 was defined as the main outcome was constructed. |
|  |  | (*b*) Describe any methods used to examine subgroups and interactions | a hierarchical model in which the occurrence of COVID-19 was defined as the main outcome was constructed. |
|  |  | (*c*) Explain how missing data were addressed | Missing data, although rare, were removed from the statistical analysis and considered a negative result for crude frequency and percentage calculations. |
|  |  | (*d*) If applicable, explain how loss to follow-up was addressed | Time at risk was calculated from data enrolment to the end of follow-up, censoring or onset of COVID-19. |
|  |  | (*e*) Describe any sensitivity analyses | A ‘fully adjusted’ multivariable model that included all the measured risk factors was fitted in a sensitivity analysis to assess the extent of residual confounding. An a priori hypothetical interaction between leprosy status and BCG status was tested in both models using likelihood ratio tests. |
| Results | | |  |
| Participants | 13* | (a) Report numbers of individuals at each stage of study—eg numbers potentially eligible, examined for eligibility, confirmed eligible, included in the study, completing follow-up, and analysed | We included 406 individuals, and during the follow-up period, 69 (16.99%) of these individuals developed RT-PCR-confirmed COVID-19 (Table 1, Fig 1, S1 Table). A total of 113 patients had active leprosy, 153 individuals were leprosy HHCs, and 140 individuals were classified as controls. |
|  |  | (b) Give reasons for non-participation at each stage | Fig 1 |
|  |  | (c) Consider use of a flow diagram | Fig 1 |
| Descriptive data | 14* | (a) Give characteristics of study participants (eg demographic, clinical, social) and information on exposures and potential confounders | S1 File |
|  |  | (b) Indicate number of participants with missing data for each variable of interest | S1 Table |
|  |  | (c) Summarise follow-up time (eg, average and total amount) | Fig 1 |
| Outcome data | 15* | Report numbers of outcome events or summary measures over time | Fig 2 |
| Main results | 16 | (*a*) Give unadjusted estimates and, if applicable, confounder-adjusted estimates and their precision (eg, 95% confidence interval). Make clear which confounders were adjusted for and why they were included | S1 File |
|  |  | (*b*) Report category boundaries when continuous variables were categorized | S1 File |
|  |  | (*c*) If relevant, consider translating estimates of relative risk into absolute risk for a meaningful time period | S1 File |
| Other analyses | 17 | Report other analyses done—eg analyses of subgroups and interactions, and sensitivity analyses | S1 File |
| Discussion | | |  |
| Key results | 18 | Summarise key results with reference to study objectives | The principal model showed that no characteristic related to leprosy or BCG vaccination acted as a risk factor for or protective factor against COVID-19. Alternatively, this model showed that previous histories of HHC with COVID-19 and diabetes were risk factors for COVID-19. |
| Limitations | 19 | Discuss limitations of the study, taking into account sources of potential bias or imprecision. Discuss both direction and magnitude of any potential bias | Although the intended sample size was achieved, and the final model showed a clinically relevant result, some limitations must be acknowledged. The existence of confounders must always be considered in observational protocols, although prospective studies are paramount for adequate clinical trial design. New trials are important to test the effects of drugs, such as clofazimine and dapsone, on COVID-19 development. |
| Interpretation | 20 | Give a cautious overall interpretation of results considering objectives, limitations, multiplicity of analyses, results from similar studies, and other relevant evidence | New trials are important to test the effects of drugs, such as clofazimine and dapsone, on COVID-19 development. A previous in vitro study reported antiviral properties of clofazimine at a 200 mg daily dose. The usual dose for the treatment of leprosy, 50 mg, may not have a suppressive effect on SARS-CoV-2. No patient used a higher dose of clofazimine. |
| Generalisability | 21 | Discuss the generalisability (external validity) of the study results | Age is also related to intense pulmonary inflammation. Other risk factors, such as obesity and hypertension, were not related to severe COVID-19. However, we must consider that the population size was not sufficient to detect these secondary outcomes. Contrary to the previous assumption, BCG vaccination was not related to COVID-19 severity. This result is consistent with recent reports. |
| Other information | | |  |
| Funding | 22 | Give the source of funding and the role of the funders for the present study and, if applicable, for the original study on which the present article is based | This study was financed in part by the Coordenação de Aperfeiçoamento de Pessoal de Nível Superior - Brasil (CAPES) - Finance Code 001. |

*Give information separately for exposed and unexposed groups.

**Note:** An Explanation and Elaboration article discusses each checklist item and gives methodological background and published examples of transparent reporting. The STROBE checklist is best used in conjunction with this article (freely available on the Web sites of PLoS Medicine at http://www.plosmedicine.org/, Annals of Internal Medicine at http://www.annals.org/, and Epidemiology at http://www.epidem.com/). Information on the STROBE Initiative is available at http://www.strobe-statement.org.
