# Supplementary material for: The influence of leprosy-related clinical and epidemiological variables in the occurrence and severity of COVID-19: A prospective real-world cohort study
Source: PLoS Negl Trop Dis. 2021 Jul 28;15(7):e0009635. doi: 10.1371/journal.pntd.0009635 (PMC8351963; doi:10.1371/journal.pntd.0009635)
Supplement: S1 File — (DOCX) [file pntd.0009635.s003.docx]

**Descriptive and univariate statistics**

Cohort characteristics (n=406)

|  |  | leprosy status | | |  |
| --- | --- | --- | --- | --- | --- |
|  |  | Control (n = 140) | Leprosy HHC (n = 153) | Active leprosy (n = 113) | P-value† |
| Age (years) |  | 36.77 (16.52) | 38.03 (16.57) | 45.60 (15.02) | <0.001 |
| Age group (years) | <18 | 3 (2.14%) | 17 (11.11%) | 5 (4.42%) | <0.001 |
|  | 18 - 29 | 62 (44.29%) | 30 (19.61%) | 13 (11.50%) |  |
|  | 30 - 39 | 18 (12.86%) | 36 (23.53%) | 20 (17.69%) |  |
|  | 40 - 49 | 14 (10.00%) | 34 (22.22%) | 25 (22.12%) |  |
|  | 50 - 59 | 28 (20.00%) | 19 (12.42%) | 26 (23.01%) |  |
|  | 60+ | 15 (10.71%) | 17 (11.11%) | 24 (21.24%) |  |
| Sex | Female | 68 (48.57%) | 79 (51.63%) | 58 (51.33%) | 0.853 |
|  | Male | 72 (51.43%) | 74 (48.37%) | 55 (48.67%) |  |
| Obesity (BMI > 29 kg/m^2^) |  | 3 (2.14%) | 21 (13.73%) | 21 (18.58%) | <0.001 |
| Hypertension |  | 17 (12.14%) | 28 (18.30%) | 25 (22.12%) | 0.102 |
| Diabetes |  | 3 (2.14%) | 4 (2.61%) | 21 (18.58%) | <0.001 |
| Smoking (current) |  | 6 (4.29%) | 44 (28.76%) | 25 (21.12%) | <0.001 |
| Alcohol dependency |  | 5 (3.57%) | 39 (25.49%) | 19 (16.81%) | <0.001 |
| Drug dependency |  | 5 (3.57%) | 39 (25.49%) | 19 (16.81%) | <0.001 |
| Previous leprosy |  | 46 (32.86%) | 9 (5.88%) | 45 (39.82%) | <0.001 |
| BCG vaccination (doses) | 0 | 11 (7.86%) | 15 (9.80%) | 45 (39.82%) | <0.001 |
|  | 1 | 107 (76.43%) | 53 (34.64%) | 47 (41.59%) |  |
|  | 2 | 22 (15.71%) | 85 (55.55%) | 21 (18.58%) |  |

Table shows frequency (%) or mean (standard deviation); n = number of patients; HHC = household contacts; BMI = body mass index; BCG = Bacillus Calmette-Guérin

† Chi-square test for categorical variables (or Fisher’s exact test if frequency ≤ 5); one-way ANOVA or Kruskal-Wallis tests for continuous variables

Ethnic characteristics of included individuals according to the Brazilian Institute of Geography and Statistics (IBGE)

| Ethnic group / National classification | Control (n = 140) | Leprosy HHC (n = 153) | Active leprosy (n = 113) |
| --- | --- | --- | --- |
| Asian (amarelo) | 2 (1.42%) | 1 (0.65%) | 2 (1.77%) |
| Black (preto) | 20 (14.29%) | 23 (15.03%) | 35 (31.97%) |
| Brown (pardo) | 44 (31.43%) | 60 (39.22%) | 38 (33.63%) |
| Indigenous (indígena) | 1 (0.71%) | 0 | 0 |
| White (branco) | 73 (52.14%) | 69 (45.10%) | 38 (33.63%) |

HHC = household contacts, Fisher’s exact test: Leprosy HHC x Control = 0.487; Leprosy x Control = 0.003; Leprosy x Leprosy HHC = 0.007.

Characteristics of active leprosy patients in the cohort by COVID-19 status (N=113)

|  |  | No COVID-19 (n = 80) | Confirmed COVID-19 (n=33) | P-value† |
| --- | --- | --- | --- | --- |
| Age (years) |  | 44.85 (15.50) | 47.42 (13.83) | 0.388 |
| Sex |  |  |  | 0.856 |
| Female |  | 42 (52.50%) | 16 (48.48%) |  |
| Male |  | 38 (47.50%) | 17 (51.51%) |  |
| Previous leprosy |  | 35 (43.75%) | 10 (30.30%) | 0.264 |
| Skin slit smear result | 0 | 51 (63.75%) | 23 (69.69%) | 0.830 |
|  | 1-4 | 18 (22.50%) | 6 (18.18%) |  |
|  | 5+ | 11 (13.75%) | 4 (12.12%) |  |
| Disability grade | 0 | 22 (27.50%) | 10 (30.30%) | 0.670 |
|  | 1 | 34 (42.50%) | 16 (48.48%) |  |
|  | 2 | 24 (30.00%) | 7 (21.21%) |  |
| Reaction | Type 1 | 19 (23.75%) | 5 (15.15%) | 0.860 |
|  | Type 2 | 8 (10.00%) | 3 (9.09%) |  |
|  | Both | 14 (17.50%) | 5 (15.15%) |  |
| Clofazimine |  | 76 (95.00%) | 31 (93.93%) | 0.630 |
| Dapsone |  | 46 (57.50%) | 16 (48.48%) | 0.504 |
| Rifampicin |  | 69 (86.25%) | 28 (84.84%) | 1.000 |
| Corticosteroids |  | 58 (72.50%) | 22 (66.66%) | 0.695 |
| Thalidomide |  | 21 (26.25%) | 5 (15.15%) | 0.230 |
| Pentoxifylline |  | 6 (7.50%) | 2 (6.06%) | 1.000 |
| BCG vaccination (doses) | 0 | 34 (42.50%) | 11 (33.33%) | 0.192 |
|  | 1 | 29 (36.25%) | 18 (54.54%) |  |
|  | 2 | 17 (21.25%) | 4 (12.12%) |  |

n = number of patients; BCG = Bacillus Calmette-Guérin.

† Chi-square test for categorical variables (or Fisher’s exact test if cell frequency ≤ 5); Student's t-test or Mann–Whitney U test for numerical variables

**Exploratory analysis**

Effect of leprosy status, BCG and COVID household contact on risk of COVID-19 with interaction between HD status and BCG status (N=406)

|  |  | n | COVID | Partially adjusted HR (95% CI)† | Fully adjusted HR (95% CI)‡ |
| --- | --- | --- | --- | --- | --- |
| BCG 0 | Controls | 11 | 3 | 1.00 | 1.00 |
|  | leprosy HHC | 15 | 4 | 0.52 (0.11-2.54) | 0.70 (0.13-3.77) |
|  | Active leprosy | 45 | 11 | 0.60 (0.16-2.26) | 0.66 (0.17-2.58) |
| BCG 1 | Controls | 107 | 4 | 1.00 | 1.00 |
|  | lerprosy HHC | 53 | 10 | 4.51 (1.31-15.6) | 4.61 (1.31-16.3) |
|  | Active leprosy | 47 | 18 | 4.85 (1.57-15.0) | 5.26 (1.67-16.6) |
| BCG 2 | Controls | 22 | 3 | 1.00 | 1.00 |
|  | leprosy HHC | 85 | 12 | 0.47 (0.12-1.88) | 0.49 (0.12-2.03) |
|  | Active leprosy | 21 | 4 | 1.20 (0.26-5.55) | 1.21 (0.26-5.73) |
| Controls | BCG 0 | 11 | 3 | 1.00 | 1.00 |
|  | BCG 1 | 107 | 4 | 0.16 (0.03-0.78) | 0.18 (0.04-0.91) |
|  | BCG 2 | 22 | 3 | 0.57 (0.11-3.00) | 0.62 (0.11-3.38) |
| leprosy HHC | BCG 0 | 15 | 4 | 1.00 | 1.00 |
|  | BCG 1 | 53 | 10 | 1.42 (0.43-4.72) | 1.20 (0.34-4.27) |
|  | BCG 2 | 85 | 12 | 0.52 (0.15-1.74) | 0.44 (0.13-1.52) |
| Active leprosy | BCG 0 | 45 | 11 | 1.00 | 1.00 |
|  | BCG 1 | 47 | 18 | 1.33 (0.60-2.92) | 1.45 (0.64-3.27) |
|  | BCG 2 | 21 | 4 | 1.15 (0.36-3.75) | 1.13 (0.33-3.81) |
| COVID-19 HHC | No | 331 | 28 | 1.00 | 1.00 |
|  | Yes | 75 | 41 | 7.55 (4.44-12.9) | 7.37 (4.30-12.6) |

HR = hazard ratio; CI = confidence interval; BCG = Bacillus Calmette–Guérin; HHC = household contact

Risk factors that met a low evidential threshold (p≤0.1) for possible associations with leprosy status and COVID-19 and that were not deemed to be on a causal pathway were carried forward to a ‘partially adjusted’ multivariable model. A ‘fully adjusted’ multivariable model that included all the measured risk factors was fitted in a sensitivity analysis to assess the extent of residual confounding.

† Adjusted for age group, hypertension, diabetes, drug abuse, previous leprosy, BCG doses, COVID-19 household contact (Likelihood ratio test p-value for interaction = 0.05)

‡ Adjusted for age group, sex, obesity, hypertension, diabetes, smoking, alcohol abuse, drug abuse, personal protection, social distancing, previous leprosy, BCG doses, COVID-19 household contact (Likelihood ratio test p-value for interaction = 0.06)

Effect of HD status, BCG and COVID household contact on risk of COVID-19 with interaction between HD status and BCG status (0 or 1 compared with 2 doses) (N=406)

|  |  | n | Confirmed  COVID-19 | Partially adjusted HR (95% CI)† | Fully adjusted HR (95% CI)‡ |
| --- | --- | --- | --- | --- | --- |
| BCG 0 or 1 | Controls | 118 | 7 | 1.00 | 1.00 |
|  | leprosy HHC | 68 | 14 | 2.54 (0.93-6.93) | 2.74 (0.98-7.72) |
|  | Active leprosy | 92 | 29 | 2.74 (1.14-6.58) | 2.86 (1.17-6.97) |
| BCG 2 | Controls | 22 | 3 | 1.00 | 1.00 |
|  | leprosy HHC | 85 | 12 | 0.47 (0.12-1.91) | 0.49 (0.12-2.01) |
|  | Active leprosy | 21 | 4 | 1.22 (0.26-5.64) | 1.20 (0.26-5.68) |
| Controls | BCG 0 or 1 | 118 | 7 | 1.00 | 1.00 |
|  | BCG 2 | 22 | 3 | 2.22 (0.57-8.69) | 2.23 (0.56-8.86) |
| leprosy HHC | BCG 0 or 1 | 68 | 14 | 1.00 | 1.00 |
|  | BCG 2 | 85 | 12 | 0.41 (0.18-0.97) | 0.40 (0.17-0.94) |
| Active leprosy | BCG 0 or 1 | 92 | 29 | 1.00 | 1.00 |
|  | BCG 2 | 21 | 4 | 0.99 (0.34-2.86) | 0.94 (0.31-2.83) |
| COVID-19 HHC | No | 331 | 28 | 1.00 | 1.00 |
|  | Yes | 75 | 41 | 7.71 (4.54-13.1) | 7.54 (4.41-12.9) |

HR = hazard ratio; CI = confidence interval; BCG = Bacillus Calmette–Guérin; HHC = household contact

Risk factors that met a low evidential threshold (p≤0.1) for possible associations with leprosy status and COVID-19 and that were not deemed to be on a causal pathway were carried forward to a ‘partially adjusted’ multivariable model. A ‘fully adjusted’ multivariable model that included all the measured risk factors was fitted in a sensitivity analysis to assess the extent of residual confounding.

† Adjusted for age group, hypertension, diabetes, drug abuse, previous leprosy, BCG doses, COVID-19 household contact (Likelihood ratio test p-value for interaction = 0.13)

‡ Adjusted for age group, sex, obesity, hypertension, diabetes, smoking, alcohol abuse, drug abuse, personal protection, social distancing, previous HD, BCG doses, COVID-19 household contact (Likelihood ratio test p-value for interaction = 0.12)

**Alternative outcomes**

The influence of possible risk factors on the occurrence of severe outcomes related to COVID-19. Data evaluated in only patients who developed COVID-19(N = 69)

|  | MILD OUTCOME | SEVERE OUTCOME | P value^†^ | Relative risk  (95%CI) |
| --- | --- | --- | --- | --- |
| Sex |  |  | 1 | 1.05  (0.44-2.49) |
| Female | 29(54;72%) | 9(56.25%) |  |  |
| Male | 24(45.28%) | 7(43.75%) |  |  |
| Age | 39.91(15.71) | 52.00(10.60) | 0.005^‡^ | 2.91‡  (1.04-8.15) |
| Leprosy | 25(47.17%) | 8(50.00%) | 1 | 1.09  (0.46-2.57) |
| Clofazimine use | 27(50.94) | 8(50.00%) | 1 | 0.97  (0.41-2.29) |
| Dapsone use | 16(30.19%) | 3(18.75%) | 0.527 | 0.61  (0.19-1.89) |
| Rifampicin use | 26(49.06%) | 8(50.00%) | 1 | 1.03  (0.44-2.43) |
| Obesity | 6(11.32%) | 3(18.75%) | 0.423 | 1.54  (0.55-4.36) |
| Hypertension | 14(26.42%) | 5(31.25%) | 0.952 | 1.19  (0.48-2.99) |
| Diabetes | 7(13.21%) | 5(31.25%) | 0.196 | 2.16  (0.92-5.07) |
| Smoking | 8(15.09%) | 2(12.50%) | 1 | 0.84  (0.22-3.16) |
| Alcohol abuse | 9(16.98%) | 2(12.50%) | 1 | 0.75  (0.19-2.86) |
| Corticosteroids use | 21(39.62%) | 10(62.50%) | 0.185 | 2.04  (0.84-4.99) |
| Thalidomide use | 8(15.09%) | 3(18.75%) | 0.708 | 1.21  (0.41-3.57) |
| Pentoxifylline use | 4(7.54%) | 1(6.25%) | 1 | 0.85  (0.14-5.20) |
| COVID-19 HHC | 30(56.60%) | 11(68.75%) | 0.564 | 1.50  (0.59-3.85) |
| Social distancing compliance | 41(76.36%) | 16(100%) | 0.086 | 7.39  (0.47-115.50) |
| BCG doses |  |  | 0.202 | 0.38  (0.09-1.50) |
| 0 | 12(22.64%) | 6(37.50%) |  |  |
| 1 | 24(45.28%) | 8(50.00%) |  |  |
| 2 | 17(32.08%) | 2(12.50%) |  |  |
| Total (n) | 53 | 16 |  |  |

Table shows frequency (%) or mean (standard deviation); CI = confidence interval; BCG = Bacillus Calmette–Guérin; HHC = household contact

†Chi-squared test for categorical variables (or Fisher’s exact test if frequency ≤ 5); Student's t-test or Mann–Whitney U test for numerical variables

‡Comparison between patients older and younger than 43 years (mean age). n = number of patients; CI = confidence interval

**Main outcome**

Unadjusted analysis of possible risk factors for the occurrence of COVID-19(N = 406)

|  | COVID-19 + | COVID-19 - | P value^†^ | P value  (survival) | Relative risk  (95%CI) | Hazard ratio |
| --- | --- | --- | --- | --- | --- | --- |
| Sex |  |  | 0.482 | 0.400 | -- | -- |
| Female | 38(55.07%) | 167(49.55%) | -- | -- | -- | 1.00 |
| Male | 31(44.93%) | 170(50.45%) | -- | -- | 0.83  (0.54-1.28) | 0.82  (0.51-1.31) |
| Age | 42.71(15.49) | 38.09(16.67) | 0.097 | 0.011‡ | 1.77‡  (1.13-2.78) | 1.011  (0.99-1.03) |
| Leprosy | 33(47.83%) | 80(23.74%) | <0.001 | <0.001 | 2.38  (1.56-3.62) | 2.59  (1.62-4.16) |
| Clofazimine use | 31(44.93%) | 76(22.55%) | <0.001 | <0.001 | 2.28  (1.49-3.46) | 2.45  (1.53-3.94) |
| Dapsone use | 16(23.19%) | 46(13.65%) | 0.068 | 0.045 | 1.68  (1.03-2.73) | 1.76  (1.01-3.07) |
| Rifampicin use | 28(40.58%) | 69(20.47%) | <0.001 | <0.001 | 2.18  (1.43-3.32) | 2.33  (1.44-3.77) |
| Leprosy HHC | 26(37.68%) | 127(37.69%) | 1 | 0.054 | 0.99  (0.64-1.56) | 1.02  (0.62-1.65) |
| Previous leprosy | 23(33.33%) | 77(22.85%) | 0.091 | 0.054 | 1.53  (0.98-2.39) | 1.63  (0.99-2.69) |
| Obesity | 9(13.04%) | 36(10.68%) | 0.719 | 0.600 | 1.20  (0.64-2.26) | 1.21  (0.59-2.43) |
| Hypertension | 19(27.53%) | 51(15.13%) | 0.021 | 0.018 | 1.82  (1.15-2.89) | 1.87  (1.10-3.18) |
| Diabetes | 12(17.39%) | 16(4.75%) | <0.001 | <0.001 | 2.84  (1.74-4.64) | 3.39  (1.82-6.32) |
| Smoking | 10(14.49%) | 65(19.29%) | 0.444 | 0.370 | 0.75  (0.40-1.39) | 0.73  (0.38-1.44) |
| Alcohol abuse | 11(15.94%) | 52(15.43%) | 1 | 0.930 | 1.033  (0.57-1.86) | 1.03  (0.54-1.96) |
| Drug abuse | 1(1.45) | 36(10.68) | 0.011 | 0.019 | 0.15  (0.02-1.03) | 0.14  (0.02-0.97) |
| Corticosteroids use | 31(44.93) | 77(22.85) | <0.001 | <0.001 | 2.25  (1.48-3.43) | 2.44  (1.52-3.92) |
| Thalidomide use | 11(15.94%) | 27(8.01%) | 0.067 | 0.038 | 1.84  (1.06-3.19) | 1.96  (1.03-3.73) |
| Pentoxifylline use | 5(7.25%) | 9(2.67%) | 0.125 | 0.037 | 2.19  (1.05-4.57) | 2.55  (1.03-6.34) |
| Adequate PPE use | 65(92.75) | 308(91.39) | 0.628 | 0.420 | 1.44  (0.56-3.69) | 1.51  (0.55-4.15) |
| COVID-19 HHC | 41(59.42) | 34(10.08) | <0.001 | <0.001 | 6.46  (4.28-9.74) | 8.96  (5.53-14.54) |
| Social distancing compliance | 57(82.61) | 277(82.19) | 1 | 0.930 | 1.02  (0.58-1.80) | 1.03  (0.55-1.92) |
| Bacillus Calmette–Guérin doses |  |  | 0.118 | 0.086 |  |  |
| 0 | 18(26.09) | 53(15.72) | -- | -- | -- | 1.00 |
| 1 | 32(46.38) | 175(51.93) | -- | -- | -- | 0.56  (0.31-0.99) |
| 2 | 19(27.54) | 109(32.34) | -- | -- | 0.83  (0.51-1.34) | 0.54  (0.28-1.02) |
| Total (n) | 69 | 337 |  |  |  |  |

Table shows frequency (%) or mean (standard deviation); n = number of patients; CI = confidence interval; HHC = household contacts; PPE = personal protective equipment.

†Chi-squared test for categorical variables (or Fisher’s exact test if frequency ≤ 5); Student's t-test or Mann–Whitney U test for numerical variables

‡Comparison between patients older and younger than 40 years (mean age)

Risk factors for COVID-19 among patients with active leprosy (N=113)

|  |  | Confirmed  COVID-19 | Unadjusted HR (95% CI) | Partially adjusted HR (95% CI)† | Fully adjusted HR (95% CI)‡ |
| --- | --- | --- | --- | --- | --- |
| Skin slit smear result | 0(n=74) | 23 | 1.00 | 1.00 | 1.00 |
|  | 1-4(n=24) | 6 | 0.81 (0.33, 1.99) | 1.46 (0.51, 4.18) | 1.71 (0.37, 7.93) |
|  | 5+(n=15) | 4 | 0.88 (0.30, 2.53) | 3.49 (0.91, 13.4) | 9.05 (1.60, 51.2) |
| Diabetes | No(n=92) | 24 | 1.00 | 1.00 | 1.00 |
|  | Yes(n=21) | 9 | 1.87 (0.87, 4.02) | 2.86 (1.12, 7.34) | 5.58 (1.29, 24.1) |
| Obesity | No(n=92) | 25 | 1.00 | 1.00 | 1.00 |
|  | Yes(n=21) | 8 | 1.50 (0.68, 3.32) | 1.74 (0.71, 4.25) | 4.67 (1.33, 16.4) |
| Thalidomide | No(n=87) | 28 | 1.00 | 1.00 | 1.00 |
|  | Yes(n=26) | 5 | 0.56 (0.22, 1.46) | 0.22 (0.06, 0.76) | 0.09 (0.01, 0.77) |
| COVID-19 HHC | No(n=84) | 14 | 1.00 | 1.00 | 1.00 |
|  | Yes(n=29) | 19 | 5.83 (2.90, 11.7) | 8.08 (3.65, 17.9) | 15.6 (4.83, 50.1) |

n = number of patients; HR = hazard ratio; CI = confidence interval; HHC = household contact

† Adjusted for age group, skin slit smear result, diabetes, obesity, thalidomide, COVID-19 household contact

‡ Adjusted for age group, sex, skin slit smear result, obesity, hypertension, diabetes, smoking, alcohol abuse, drug abuse, personal protection equipment use, social distancing, previous leprosy, Bacillus Calmette-Guérin doses, COVID-19 household contact, disability grade, leprosy reaction, clofazimine use, dapsone use, rifampicin use, corticosteroids use, thalidomide use, pentoxifylline use.
