# Supplementary material for: The influence of leprosy-related clinical and epidemiological variables in the occurrence and severity of COVID-19: A prospective real-world cohort study
Source: PLoS Negl Trop Dis. 2021 Jul 28;15(7):e0009635. doi: 10.1371/journal.pntd.0009635 (PMC8351963; doi:10.1371/journal.pntd.0009635)
Supplement: Attachment — Submitted filename: ANSWER TO REVIEWERS.docx [file pntd.0009635.s004.docx]

Dear Editors and Reviewers,

Thank you very much for the detailed and fast review of the present manuscript. We were happy to hear all of the compliments about the importance of the present topic and that no significant methodological limitations were noted. We agree that the complexity of the variables included requires a better explanation of the objectives. We clarified all of the questions from the reviewers. Please find below our point-by-point answers to the reviewers’ questions.

Sincerely,

**Corresponding Author:**

Ciro Martins Gomes, MD, PhD, Hospital Universitário de Brasília SGAN 605, Av. L2 Norte. CEP: 70910-900, Brasília, Distrito Federal, Brazil; Phone: [+]55 (61)9155-4039. Fax: [+]55 (61)3225-6799. email: cirogomes@unb.br.

**Responses to the Reviewers’ Questions**

**Reviewer #1: The experimental design includes a prospective cohort study in which the main exposure was 2 previous BCG vaccinations, and the main outcome was COVID-19 infection. Also, secondary exposures were considered: BCG status (0, 1 or 2 doses) and leprosy status (patient, LHC or controls). This experimental design is quite interesting, but the complexity of the variables involved requires great caution when interpreting the data. In my view, the experimental design needs to be better explained. It is essential to clarify the following points:**

Thank you for the compliments related to the relevance of our experimental design. We agree that this is a complex study and that better explanations of all analyses and results must be provided. We performed all requests. Please let us know if any additional corrections are necessary.

**1. In the introduction, it was said that the aim of the study was to evaluate the effect of 2 doses of BCG on the occurrence of COVID-19. However, other approaches played a leading role in the methods and results, to test a lot of risk/protection factors to covid-19 outcome in leprosy patients. It is not consistent with the title of the paper initial proposed objective. The exploratory analysis and the results presented go far beyond what is presented in the title of the article, making it unsuitable for the work. I would like to suggest that the title as well as the excerpts relevant to the objective of the work be rewritten.**

We agree that defining objectives may be a subjective task in observational studies. As agreed by reviewers #2 and #3, we previously limited the objectives to main risk factors and main outcomes. This strategy was chosen to show readers that we respected a pre-designed protocol and that no data manipulation or interim analysis was performed.

However, we agree that our article examines a set of important information related to COVID-19. Consistent with the recommendation of reviewer #1, we made substantial changes in the title **(Page 1, lines 2 and 3)** and in reporting the objectives **(Page 6, lines 124-128)**, and we clearly stated the main risk factors and outcomes, as mentioned by reviewers #2 and #3, to validate our statistical strategy,

**2.      In the Methods section it was not clear what were the inclusion criteria for the Cohort study. It is essential to clarify these criteria for a better understanding of the experimental design. A flowchart detailing the experimental design and inclusion/exclusion criteria and criteria would be helpful.**

We included a clear explanation of the inclusion criteria that consisted of all consecutive patients referred to the Leprosy Outpatient Service of Brasília University Hospital, University of Brasília, Brazil population, which included patients with active leprosy, household contacts of patients with leprosy and patients with clinical conditions with a differential diagnosis of leprosy (controls) **(Page 7, lines 152-155)**. Figure 1 was replaced with a study flowchart following the STROBE instructions.

**3.      The strategy for defining the experimental size appeared to be for convenience, depending on the number of leprosy patients recruited in the period. It is important to clarify which method was used to calculate statistical power, and which parameters were used in the analysis.**

We better clarified in the text that we did not use a convenience sample. We used a referenced sample size calculation before the recruitment phase. Convenience samples are not sufficient for warranting internal or external validation (generalizability) of results. It is especially important to show readers that our methods, based on existing literature, warrant internal + external validation for the BCG influence in the occurrence of COVID-19, and that the study also warrants internal validation related to our population after the cohort of patients referred to the leprosy outpatient clinic were evaluated **(Page 11, lines 237-249)**.

Considering the remaining variables, we agree, as noted by reviewers #1 and #2, that our sample size is limited by the scarcity of previous data in the literature. However, we believe that our study is a scientific advance for future trials and observational studies. It provides data for sample size calculations for all of the variables described considering univariate and regression models.

The issues mentioned by reviewers #1 and #2 are better explained in the methods section. We divided sample size calculation procedures by the existing risk factors/outcomes and not by the main objective, consistent with the recommendations.

**4.      The authors do not clearly define the group "active leprosy". What is the distribution regarding the clinical forms of leprosy? Were patients at any stage of treatment included?**

As requested, a clear explanation of the criteria used to define “active leprosy” and treatment phases was included. The clinical forms of leprosy are also more clearly defined in the results section with the definitions used for this classification **(Page 8, lines 166 and 167)**.

**5.      The Brazilian population has a high degree of miscegenation. Population information about ancestry (or even race/ethnicity) was not available in the text. It would be important to consider these variables as possible confounders influencing the COVID-19 and leprosy outcomes, and if possible, reanalyze the data including this variable in the adjusted model.**

As requested, we included additional data related to ethnical, social and economic factors. Detailed data on ethnic groups and the frequency of unemployment are included in Supplementary file 1. We also included a detailed explanation about the necessity of future detailed studies relating economic and social factors to coinfection with COVID-19 and leprosy. Unfortunately, the high variability of these data made the inclusion of this information in the multivariate model unfeasible **(Page 19, lines 317-325)**.

**6.      How were defined the categories of these following variables: use of personal protective equipment (PPE) against SARS-CoV-2 infection (face mask, alcohol gel, frequent hand washing) and adoption of social distancing behaviours? It is important to explain better in the methodology.**

As requested, definitions of the mentioned variables are clearer in the text. The variables were verified during consultation and defined according to local recommendations as follows **(Pages 8 and 9, lines 180-185)**:

“Personal protection against COVID-19 was defined according to the following criteria: 1. Adequate personal protection: Patients reported having received adequate orientation and acting in full compliance with recommendations related to the use of masks in public environments, alcohol gel and frequent hand washing; 2. Social distancing: Full compliance with local recommendations and local liberations and a complete lack of participation in labour or leisure activities not allowed by current local decrees.”

**7.      For the statistical analysis the authors have used the R environment, but they mentioned only the R Studio. It is important to cite properly the R environment (R project) and all packages used.**

As requested, we included the citation of the referred environments and packages used, as recommended by the developers **(Pages 9 and 10, lines 200-232)**.

**Reviewer #2: The manuscript has a clear objective, is related to the theme and hypothesis to be tested, and has an appropriate research design to respond to the proposed objective. The population is described and appropriate for what is being analyzed by the researcher. I believe that the sample is adequate, however the presence of sample calculus was not observed. Statistical analysis are adequate to what is proposed in the study, however it is necessary to better describe the analysis of the data contained in the tables, adequate ethical precepts.**

As requested by reviewed #1, a better explanation of our sample size approach was included and clearly related to each variable and analysis. Detailed legends of statistical analyses are explained in each table with additional information related to the ethical clearance of the study **(Page 11, lines234-247)**.

**Reviewer #3: The methodology section is well defined and nicely described in the study**

Thank you very much.

**--------------------


Reviewer #1: 1. The authors mentioned that “409 patients were included in the study”. This can be confusing to the reader, since leprosy HHC and controls were also involved in the study. I suggest using another term, such as individuals.**

We replaced the word “patients” with “individuals” as requested and used the word “patients” to refer only to individuals with active leprosy cases.

**2.      The authors begin the results with the characterization of the population and then address secondary exposures. I would like to suggest that, once the main exposures of the work are clearly defined, that they come first in the order of results.**

As requested, we first described results related to the main exposure/outcome and moved exploratory analyses and additional outcomes to the end of the discussion section (**S1 File)**.

**3.      In Brazil, the Ministry of Health recommends applying the second dose of BCG in household contacts of patients with leprosy. The results in Table 1 show this, where 55.55% of the leprosy HHC group received 2 doses of BCG. As expected, the control group had a much lower percentage of the 2 doses of BCG (15,71). In fact, this constitutes a huge bias for analyzes that assess the status of leprosy as a risk factor for COVID-19. If the authors made adjustments to the analyzes in order to correct for the influence of confounders, I believe that the uncorrected results should be omitted from the main text (they can be placed as supplementary) and only the adjusted RR and HR values should be presented.**

We totally agree that univariate analysis is a delicate method for observational studies. To call the attention to our adjusted analysis, as suggested, we moved most details related to the univariate analyses to the supplementary material (**S1 File)**.

**4.      Table 3: It would be interesting to also show the p-values associated with the respective HR values (I suggest doing this for all tables). Some confidence interval values appear to be associated with borderline values of statistical significance.**

As requested, p-values were included in Table 3, which was renamed Table 2. Consistent with reviewer #2’s recommendation, we included all tables related to exploratory evaluations containing multivariate analyses using statistical cut-offs as supplementary files **(Table 2)**.

**5.      The Table 5 presents possible risk factors (diabetes, obesity and thalidomide treatment, for example) for covid-19 in leprosy patients. It was not clear in the text that this approach was within the objectives of the work. From this cut-off, the sample size was 113 patients, reflecting some high confidence interval values. Do the authors consider this N sufficient to infer about these associations? The authors might consider placing this table as supplementary material.**

We agree that the present sample size is not sufficient for any objective conclusion. However, subgroup analysis is a recognized alternative for the evaluation of confounders. This type of analysis is discussed in the methods section, and the referred table was placed in the supplementary file as requested (**S1 File)**.

**Reviewer #2: The analysis follows the presented analysis plan. As for the results, I suggest that the information that does not appear in the tables be better described, but that need to be identified so that the data can be replicated by other studies and is better understood by the reader. In the relevant tables, I suggest adding, in the description, the presence of the patient's assessment in more than one variable. It would be interesting if the variables and their respective tests were identified in the footnotes of the tables for a better understanding of the reader.**

We agree that multivariate models are difficult to represent in tables and text. Consistent with recommendations of reviewer #1, we focused on the main outcome analysis in the main document and moved exploratory and subgroup analyses to the supplementary file. This resulted in simpler methods reporting and simpler data presentation. Crude data was also included in a supplementary table to allow visual examination of simultaneous occurrences as requested. The table legends are clearer and represent the analyses of multiple variables.

**Reviewer #3: Result section is nicely presented in the study.**

Thank you very much.

**Reviewer #1: 1. In the discussion, it was hypothesized that leprosy would favor the risk of covid-19 due to immunosuppression, however TT leprosy patients, for example, may present a certain degree of cellular immune response. It would be essential to consider the complexity of the immune response to leprosy within this proposed hypothesis.**

As requested, we added information to the statement related to leprosy immunity, including possible differences related to leprosy forms. We also noted limitations for this evaluation due to the sample size of active leprosy cases **(Page 20, lines 352-353)**.

**2.      In which aspects related to COVID-19 infection in leprosy patients can the results contribute? The conclusion of the work focuses on the hypothesis of the influence of economic and social factors on vulnerability to COVID-19 in leprosy patients, but these data were not tested in the work. It would be interesting to conclude the article based on the results from the work, and its applicability.**

As requested, we included interesting data related to ethnic, social and economic characteristics of leprosy patients. Unemployment rate and ethnic group distribution in leprosy cases favours the assumption of social vulnerability related to the coinfection. However, we agree that conclusions must be based on objective results. We moderated all conclusions purely based on subjective assumptions **(Page 21, lines 363-376)**.

**Reviewer #2: The conclusion is addressed within the discussion topic, which is allowed by the journal, the considerations corroborate the study data. The existence of limitations was informed, however I felt the need for them to be better described. The authors discuss the data clearly and objectively, favoring the understanding of the topic under study and addressing its relevance to public health.**

Consistent with reviewer #1 suggestions, we improved the conclusion section and focused on the objective findings of our article.

**Reviewer #3: Very good and given key message for SARS-CV-2 global Pandemic situation for leprosy control program.**

Thank you very much.

**Reviewer #1: 1. I think that the Figure 1 is unnecessary. The Fig. 1 is only showing the time to measured outcomes, which could be explained in the text.**

As suggested, we removed Figure 1 from the article and replaced it with a study flowchart.

**2.      Table 2: please correct: “Personal protection 125 125(89.29%)”**

The correction was performed as requested.

**3.      The Table 4 could be omitted from the main text, and included only in the supplementary material, since none of the results presented in it showed statistical significance.**

We moved Table 4 to the supplementary material as requested.

**4.      The figures are not in good image quality. I indicate that the definition of the figures is improved.**

We reviewed the quality of all images and provided images in high-resolution standards following the PLOS NTD recommendations.

**5.      Some sentences in the text are difficult to interpret. I suggest a good review of the English language.**

As requested, we reviewed all sentences to improve text didactics. The text was also submitted to a professional editing service.

**Reviewer #2: It was observed in table 4 in the items clofazimine and thalidomide, a p value different from the analyzed calculation, which needs to be revised by the author, as the information in the footer referring to Fisher's exact test I ask it would be cellular or expected frequency?**

The small discrepancy in p values, which was not capable of reversing the statistical significance, was due to our management of data loss. In this specific table, we considered losses as negative values for percentages but did not include these values in statistical analyses. This methodology is better explained in the methods section.

**Reviewer #3: No required**

**--------------------


Reviewer #1: Dear authors,
The manuscript entitled “Leprosy during the COVID-19 pandemic as a model for assessing the effects of Bacillus Calmette–Guérin vaccine and clofazimine and dapsone therapy on SARS-CoV-2 infection: A prospective real-world cohort study” by Cerqueira et al., proposes to investigate the risk for COVID-19 development in leprosy patients, and to evaluate the effect of vaccination by BCG, in a sample from Brazil. The topic is extremely relevant to public health, considering the pandemic COVID-19 period, and the need for knowledge and contributions to contain the COVID-19 new cases and dissemination. The relationship between COVID-19 and leprosy arouses a lot of interest due mainly to the immunological complexity involving both diseases. The authors performed a 14-month prospective real-world cohort study and they investigated possible risk factors to COVID-19 outcome. They have included leprosy patients, household contacts and controls. The results showed that no characteristic related to leprosy or BCG vaccination acted as risk or protective factor against COVID-19. The methodology was well conducted; however, I have some observations and suggestions mainly regarding the experimental design and results. I believe that the writing of the manuscript needs a lot of improvement to clarify the rationale involved in the experimental design as well as the proposed objectives. I hope that the modifications suggested should contribute to improve the quality of the manuscript.**

Your suggestions clearly improved the quality of our report. We hope we have properly addressed all of the requests.

**Reviewer #2: Study of interest, rich in important information for clinical practice, favors and expands knowledge about SARS-Cov-2 and its association with leprosy and with BCG vaccination, and regardless of the result, it is already an interesting and necessary proposal. This is the strength of the study, especially at this time we are experiencing. The weak point is related to the questions and suggestions already mentioned in the previous topics.**

Your suggestions clearly improved the quality of our report. We hope we have properly addressed all of the requests. We gave special attention to the discussion of study limitations.

**Reviewer #3: The manuscript Number PNTD-D-21-00714 entitled "Leprosy during the COVID-19 pandemic as a model for assessing the effects of Bacillus Calmette–Guérin vaccine and clofazimine and dapsone therapy on SARS-CoV-2 infection: A prospective real-world cohort study". This is very good study, which showing the risk of SARS-CoV-2 infection and its relation to BCG vaccine and clofazimine and dapsone treatment. The manuscript is well written and nicely presented. I would like the authors to consider the following points before it can be considered.**

Thank you very much. All corrections were made, and all questions were answered in detail.

**In table 2: Personal protection 125 125 (89.29%) ?**

This correction was made as requested.

**Secondary exposures of interest: use of PPE (Tables 1 e 2) ?**

The abbreviation “PPE” was removed from most tables and texts. An adequate explanation was included in the remaining table legends.

**--------------------

PLOS authors have the option to publish the peer review history of their article (<a href="**[**https://journals.plos.org/plosntds/s/editorial-and-peer-review-process#loc-peer-review-history**](https://journals.plos.org/plosntds/s/editorial-and-peer-review-process#loc-peer-review-history)**" target="_blank">what does this mean?</a>). If published, this will include your full peer review and any attached files.<br><br>

If you choose “no”, your identity will remain anonymous but your review may still be made public.<br><br>

<b>Do you want your identity to be public for this peer review?</b> For information about this choice, including consent withdrawal, please see our <a href="**[**https://www.plos.org/privacy-policy**](https://www.plos.org/privacy-policy)**" target="_blank">Privacy Policy</a>.

Reviewer #1: No

Reviewer #2: No

Reviewer #3: No**
